# Editorial: Multi-modal learning with large-scale models

**DOI:** 10.3389/fnbot.2026.1816779

**Published:** 2026-03-13

**Authors:** Xianmin Wang, Jing Li

**Affiliations:** 1 Institute of Artificial Intelligence, Guangzhou University, Guangzhou, China; 2 School of Computer Science and Cyber Engineering, Guangzhou University, Guangzhou, China

**Keywords:** autonomous systems, large-scale models, multi-modal learning, neurorobotics, reinforcement learning

The integration of multi-modal learning with large-scale models has become a transformative force in the fields of artificial intelligence and neurorobotics. Human perception naturally relies on the seamless fusion of various sensory inputs—visual, auditory, tactile, and beyond—to navigate and understand complex environments. Replicating this holistic capability in intelligent systems has historically been constrained by computational limitations and the difficulty of aligning heterogeneous data. However, the advent of large-scale models has shifted the paradigm, offering unprecedented capacity to process, align, and fuse multi-modal data. This Research Topic, “*Multi-modal Learning with Large-scale Models*,” aims to explore the cutting edge of these architectures, emphasizing their applications across robotic perception, autonomous navigation, human-robot interaction, and creative generation. The seven articles gathered in this Research Topic illustrate how multi-modal large-scale models are bridging the gap between isolated data streams and unified machine cognition.

A central theme of this Research Topic is the enhancement of autonomous systems through advanced multi-modal perception and reinforcement learning. In unstructured and dynamic environments, single-modality sensors often fall short. Addressing this vulnerability, Almujally et al. introduce Deep Fused Networks (DFN) for contextual scene learning. By merging multi-object detection and semantic analysis using RGB-D data, their framework significantly improves resilience and context awareness for autonomous vehicles and robotics. Similarly targeting dynamic operations, Li et al. focus on the aerial domain with NavBLIP, a visual-language model designed for Unmanned Aerial Vehicles (UAVs). NavBLIP integrates transfer learning and a Nuisance-Invariant Multimodal Feature Extraction (NIMFE) module, allowing UAVs to disentangle task-relevant features from environmental noise such as varying weather or altitudes, thereby optimizing real-time navigation and object detection.

Transitioning to agricultural robotics, Mao et al. present an enhanced multi-modal target detection method, YOLOv5-Litchi, tailored for complex natural scenes. By optimizing detection heads and loss functions, their model successfully addresses challenges like dense occlusion and small target identification, providing vital technical support for automated crop yield estimation in unstructured outdoor settings. For ground-based mobile robots, global optimal path planning remains a computational hurdle. Jing and Weiya tackle this by proposing RL-QPSO Net, which ingeniously couples Deep Reinforcement Learning with Quantum-behaved Particle Swarm Optimization. This dual-mechanism approach utilizes quantum mechanics principles to escape local optima during path planning, while reinforcement learning dynamically adjusts strategies in response to environmental feedback, ensuring adaptability in high-dimensional settings.

Beyond navigation and perception, large-scale multi-modal models are demonstrating profound capabilities in education, human-computer interaction, and creative expression. Chang et al. revolutionize traditional music education by proposing MusicARLtrans Net, an intelligent interactive agent. Their system leverages an Align Before Fuse (ALBEF) architecture and Speech-to-Text technology to seamlessly integrate auditory, visual, and textual data, employing reinforcement learning to provide personalized, real-time teaching feedback. In the realm of language acquisition, Wang introduces ETG-ALtrans, a multi-modal robot-assisted framework for English writing guidance and error correction. By combining the ALBEF model with visual feature extraction from VGG19 networks, and refining correction strategies via reinforcement learning, the model achieves a precise alignment of image and text context, greatly outperforming traditional language models in catering to personalized learner needs.

Exploring the intersection of physiological signals and creative generation, Jiang et al. present a pioneering method for electroencephalography (EEG)-driven emotive music generation. The authors tackle the complex mapping between continuous brainwave signals and musical elements by employing clustering algorithms to establish discrete representations. Utilizing a Transformer architecture equipped with multi-head attention and positional encoding, their model translates human neural activity into emotionally coherent musical compositions, showcasing the profound potential of multi-modal architectures in bridging the cognitive-creative divide.

Collectively, the contributions within this Research Topic highlight the transformative impact of multi-modal large-scale models across a spectrum of neurorobotic applications. By moving beyond isolated sensory processing, these models enable machines to interpret the world with a depth and context that more closely mimics human cognition. Whether deployed in autonomous drones navigating turbulent skies, agricultural robots identifying occluded crops, or interactive agents providing personalized educational guidance, the fusion of multiple data modalities with robust learning algorithms is paving the way for next-generation intelligent systems. As we look to the future, we anticipate that the continued evolution of large-scale architectures will further resolve current limitations in computational efficiency, data alignment, and real-time adaptability. We extend our sincere gratitude to all the contributing authors and reviewers for their dedication and insightful work, which has made this Research Topic a valuable resource for researchers and practitioners alike.

